# Deep learning for computational biology

**DOI:** 10.15252/msb.20156651

**Published:** 2016-07-29

**Authors:** Christof Angermueller, Tanel Pärnamaa, Leopold Parts, Oliver Stegle

**Affiliations:** ^1^ European Molecular Biology LaboratoryEuropean Bioinformatics InstituteWellcome Trust Genome CampusHinxtonCambridgeUK; ^2^ Department of Computer ScienceUniversity of TartuTartuEstonia; ^3^ Wellcome Trust Sanger InstituteWellcome Trust Genome CampusHinxtonCambridgeUK

**Keywords:** cellular imaging, computational biology, deep learning, machine learning, regulatory genomics, Computational Biology

## Abstract

Technological advances in genomics and imaging have led to an explosion of molecular and cellular profiling data from large numbers of samples. This rapid increase in biological data dimension and acquisition rate is challenging conventional analysis strategies. Modern machine learning methods, such as deep learning, promise to leverage very large data sets for finding hidden structure within them, and for making accurate predictions. In this review, we discuss applications of this new breed of analysis approaches in regulatory genomics and cellular imaging. We provide background of what deep learning is, and the settings in which it can be successfully applied to derive biological insights. In addition to presenting specific applications and providing tips for practical use, we also highlight possible pitfalls and limitations to guide computational biologists when and how to make the most use of this new technology.

## Introduction

Machine learning methods are general‐purpose approaches to learn functional relationships from data without the need to define them *a priori* (Hastie *et al*, 2005; Murphy, 2012; Michalski *et al*, 2013). In computational biology, their appeal is the ability to derive predictive models without a need for strong assumptions about underlying mechanisms, which are frequently unknown or insufficiently defined. As a case in point, the most accurate prediction of gene expression levels is currently made from a broad set of epigenetic features using sparse linear models (Karlic *et al*, 2010; Cheng *et al*, 2011) or random forests (Li *et al*, 2015); how the selected features determine the transcript levels remains an active research topic. Predictions in genomics (Libbrecht & Noble, 2015; Märtens *et al*, 2016), proteomics (Swan *et al*, 2013), metabolomics (Kell, 2005) or sensitivity to compounds (Eduati *et al*, 2015) all rely on machine learning approaches as a key ingredient.

**Figure 1:**
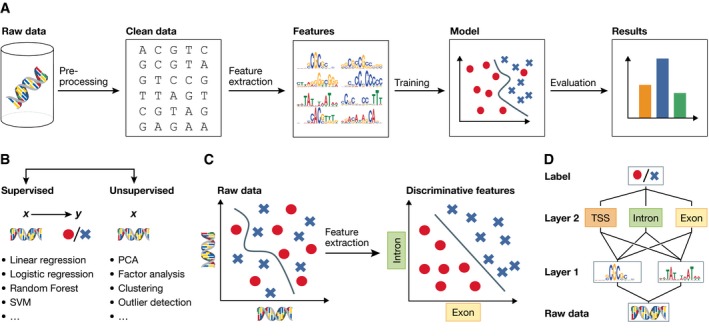
Machine learning and representation learning (A) The classical machine learning workflow can be broken down into four steps: data pre‐processing, feature extraction, model learning and model evaluation. (B) Supervised machine learning methods relate input features *x* to an output label *y*, whereas unsupervised method learns factors about *x* without observed labels. (C) Raw input data are often high‐dimensional and related to the corresponding label in a complicated way, which is challenging for many classical machine learning algorithms (left plot). Alternatively, higher‐level features extracted using a deep model may be able to better discriminate between classes (right plot). (D) Deep networks use a hierarchical structure to learn increasingly abstract feature representations from the raw data.

Most of these applications can be described within the canonical machine learning workflow, which involves four steps: data cleaning and pre‐processing, feature extraction, model fitting and evaluation (Fig 1A). It is customary to denote one data sample, including all covariates and features as *input x* (usually a vector of numbers), and label it with its response variable or *output* value *y* (usually a single number) when available.

A supervised machine learning model aims to learn a function *f*(*x*) = *y* from a list of training pairs (*x*_1_,*y*_1_), (*x*_2_,*y*_2_), … for which data are recorded (Fig 1B). One typical application in biology is to predict the viability of a cancer cell line when exposed to a chosen drug (Menden *et al*, 2013; Eduati *et al*, 2015). The input features (*x*) would capture somatic sequence variants of the cell line, chemical make‐up of the drug and its concentration, which together with the measured viability (output label *y*) can be used to train a support vector machine, a random forest classifier or a related method (functional relationship *f*). Given a new cell line (unlabelled data sample x*) in the future, the learnt function predicts its survival (output label y*) by calculating *f*(x*), even if *f* resembles more of a black box, and its inner workings of why particular mutation combinations influence cell growth are not easily interpreted. Both regression (where *y* is a real number) and classification (where *y* is a categorical class label) can be viewed in this way. As a counterpart, unsupervised machine learning approaches aim to discover patterns from the data samples *x* themselves, without the need for output labels *y*. Methods such as clustering, principal component analysis and outlier detection are typical examples of unsupervised models applied to biological data.

The inputs *x*, calculated from the raw data, represent what the model “sees about the world”, and their choice is highly problem‐specific (Fig 1C). Deriving most informative features is essential for performance, but the process can be labour‐intensive and requires domain knowledge. This bottleneck is especially limiting for high‐dimensional data; even computational feature selection methods do not scale to assess the utility of the vast number of possible input combinations. A major recent advance in machine learning is automating this critical step by learning a suitable representation of the data with *deep artificial neural networks* (Bengio *et al*, 2013; LeCun *et al*, 2015; Schmidhuber, 2015) (Fig 1D). Briefly, a deep neural network takes the raw data at the lowest (input) layer and transforms them into increasingly abstract feature representations by successively combining outputs from the preceding layer in a data‐driven manner, encapsulating highly complicated functions in the process (Box 1). Deep learning is now one of the most active fields in machine learning and has been shown to improve performance in image and speech recognition (Hinton *et al*, 2012; Krizhevsky *et al*, 2012; Graves *et al*, 2013; Zeiler & Fergus, 2014; Deng & Togneri, 2015), natural language understanding (Bahdanau *et al*, 2014; Sutskever *et al*, 2014; Lipton, 2015; Xiong *et al*, 2016), and most recently, in computational biology (Eickholt & Cheng, 2013; Dahl *et al*, 2014; Leung *et al*, 2014; Sønderby & Winther, 2014; Alipanahi *et al*, 2015; Wang *et al*, 2015; Zhou & Troyanskaya, 2015; Kelley *et al*, 2016).

**Box 1: Artificial Neural Network**

An artificial neural network, initially inspired by neural networks in the brain (McCulloch & Pitts, 1943; Farley & Clark, 1954; Rosenblatt, 1958), consists of layers of interconnected compute units (neurons). The depth of a neural network corresponds to the number of hidden layers, and the width to the maximum number of neurons in one of its layers. As it became possible to train networks with larger numbers of hidden layers, artificial neural networks were rebranded to “deep networks”.

In the canonical configuration, the network receives data in an input layer, which are then transformed in a nonlinear way through multiple hidden layers, before final outputs are computed in the output layer (panel A). Neurons in a hidden or output layer are connected to all neurons of the previous layer. Each neuron computes a weighted sum of its inputs and applies a nonlinear activation function to calculate its output *f*(*x*) (panel B). The most popular activation function is the rectified linear unit (ReLU; panel B) that thresholds negative signals to 0 and passes through positive signal. This type of activation function allows faster learning compared to alternatives (e.g. sigmoid or tanh unit) (Glorot *et al*, 2011).

The weights *w* ^(i)^ between neurons are free parameters that capture the model's representation of the data and are learned from input/output samples. Learning minimizes a loss function *L*(*w*) that measures the fit of the model output to the true label of a sample (panel A, bottom). This minimization is challenging, since the loss function is high‐dimensional and non‐convex, similar to a landscape with many hills and valleys (panel C). It took several decades before the *backward propagation algorithm* was first applied to compute a loss function gradient via chain rule for derivatives (Rumelhart *et al*, 1988), ultimately enabling efficient training of neural networks using stochastic gradient descent. During learning, the predicted label is compared with the true label to compute a loss for the current set of model weights. The loss is then backward propagated through the network to compute the gradients of the loss function and update (panel A). The loss function *L(w)* is typically optimized using gradient‐based descent. In each step, the current weight vector (red dot) is moved along the direction of steepest descent *dw* (direction arrow) by learning rate η (length of vector). Decaying the learning rate over time allows to explore different domains of the loss function by jumping over valleys at the beginning of the training (left side) and fine‐tune parameters with smaller learning rates in later stages of the model training. While learning in deep neural networks remains an active area of research, existing software packages (Table 1) can already be applied without knowledge of the mathematical details involved.

Alternative architectures to such fully connected feedforward networks have been developed for specific applications, which differ in the way neurons are arranged. These include convolutional neural networks, which are widely used for modelling images (Box 2), recurrent neural networks for sequential data (Sutskever, 2013; Lipton, 2015), or restricted Boltzmann machines (Salakhutdinov & Larochelle, 2010; Hinton, 2012) and autoencoders (Hinton & Salakhutdinov, 2006; Alain *et al*, 2012; Kingma & Welling, 2013) for unsupervised learning. The choice of network architecture and other parameters can be made in a data‐driven and objective way by assessing the model performance on a validation data set.

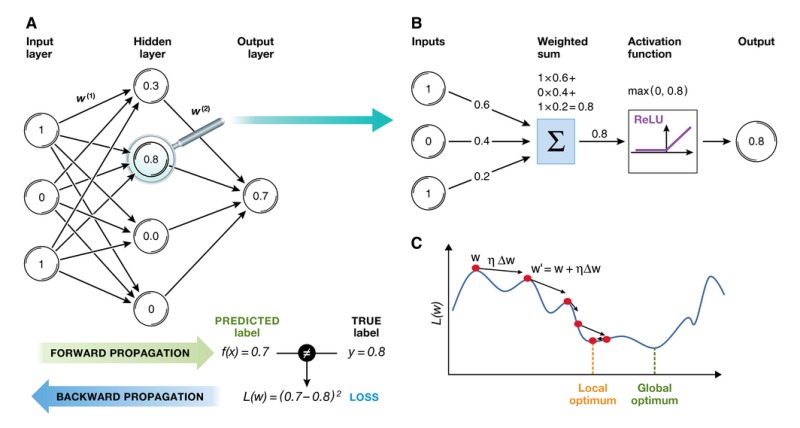

The potential of deep learning in high‐throughput biology is clear: in principle, it allows to better exploit the availability of increasingly large and high‐dimensional data sets (e.g. from DNA sequencing, RNA measurements, flow cytometry or automated microscopy) by training complex networks with multiple layers that capture their internal structure (Fig 1C and D). The learned networks discover high‐level features, improve performance over traditional models, increase interpretability and provide additional understanding about the structure of the biological data.

In this review, we discuss recent and forthcoming applications of deep learning, with a focus on applications in regulatory genomics and biological image analysis. The goal of this review was not to provide comprehensive background on all technical details, which can be found in the more specialized literature (Bengio, 2012; Bengio *et al*, 2013; Deng, 2014; Schmidhuber, 2015; Goodfellow *et al*, 2016). Instead, we aimed to provide practical pointers and the necessary background to get started with deep architectures, review current software solutions and give recommendations for applying them to data. The applications we cover are deliberately broad to illustrate differences and commonalities between approaches; reviews focusing on specific domains can be found elsewhere (Park & Kellis, 2015; Gawehn *et al*, 2016; Leung *et al*, 2016; Mamoshina *et al*, 2016). Finally, we discuss both the potential and possible pitfalls of deep learning and contrast these methods to traditional machine learning and classical statistical analysis approaches.

## Deep learning for regulatory genomics

**Figure 2:**
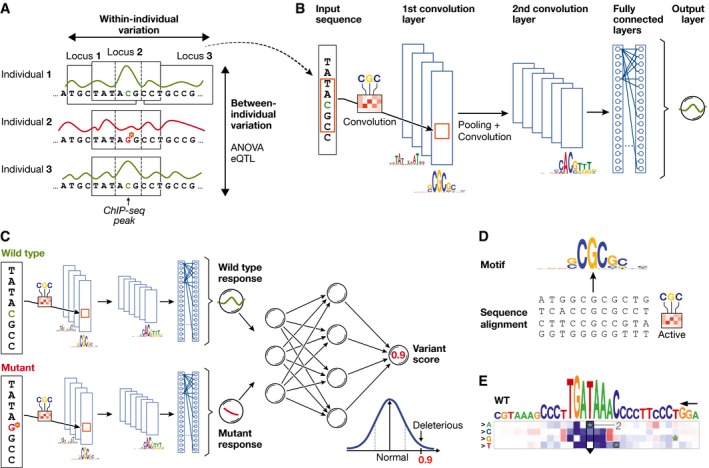
Principles of using neural networks for predicting molecular traits from DNA sequence (A) DNA sequence and the molecular response variable along the genome for three individuals. Conventional approaches in regulatory genomics consider variations between individuals, whereas deep learning allows exploiting intra‐individual variations by tiling the genome into sequence DNA windows centred on individual traits, resulting in large training data sets from a single sample. (B) One‐dimensional convolutional neural network for predicting a molecular trait from the raw DNA sequence in a window. Filters of the first convolutional layer (example shown on the edge) scan for motifs in the input sequence. Subsequent pooling reduces the input dimension, and additional convolutional layers can model interactions between motifs in the previous layer. (C) Response variable predicted by the neural network shown in (B) for a wild‐type and mutant sequence is used as input to an additional neural network that predicts a variant score and allows to discriminate normal from deleterious variants. (D) Visualization of a convolutional filter by aligning genetic sequences that maximally activate the filter and creating a sequence motif. (E) Mutation map of a sequence window. Rows correspond to the four possible base pair substitutions, columns to sequence positions. The predicted impact of any sequence change is colour‐coded. Letters on top denote the wild‐type sequence with the height of each nucleotide denoting the maximum effect across mutations (figure panel adapted from Alipanahi *et al*, 2015).

Conventional approaches for regulatory genomics relate sequence variation to changes in molecular traits. One approach is to leverage variation between genetically diverse individuals to map quantitative trait loci (QTL). This principle has been applied to identify regulatory variants that affect gene expression levels (Montgomery *et al*, 2010; Pickrell *et al*, 2010), DNA methylation (Gibbs *et al*, 2010; Bell *et al*, 2011), histone marks (Grubert *et al*, 2015; Waszak *et al*, 2015) and proteome variation (Vincent *et al*, 2010; Albert *et al*, 2014; Parts *et al*, 2014; Battle *et al*, 2015) (Fig 2A). Better statistical methods have helped to increase the power to detect regulatory QTL (Kang *et al*, 2008; Stegle *et al*, 2010; Parts *et al*, 2011; Rakitsch & Stegle, 2016); however, any mapping approach is intrinsically limited to variation that is present in the training population. Thus, studying the effects of rare mutations in particular requires data sets with very large sample size.

An alternative is to train models that use variation between regions within a genome (Fig 2A). Splitting the sequence into windows centred on the trait of interest gives rise to tens of thousands of training examples for most molecular traits even when using a single individual. Even with large data sets, predicting molecular traits from DNA sequence is challenging due to multiple layers of abstraction between the effect of individual DNA variants and the trait of interest, as well as the dependence of the molecular traits on a broad sequence context and interactions with distal regulatory elements.

The value of deep neural networks in this context is twofold. First, classical machine learning methods cannot operate on the sequence directly, and thus require pre‐defining features that can be extracted from the sequence based on prior knowledge (e.g. the presence or absence of single‐nucleotide variants (SNVs), k‐mer frequencies, motif occurrences, conservation, known regulatory variants or structural elements). Deep neural networks can help circumventing the manual extraction of features by learning them from data. Second, because of their representational richness, they can capture nonlinear dependencies in the sequence and interaction effects and span wider sequence context at multiple genomic scales. Attesting to their utility, deep neural networks have been successfully applied to predict splicing activity (Leung *et al*, 2014; Xiong *et al*, 2015), specificities of DNA‐ and RNA‐binding proteins (Alipanahi *et al*, 2015) or epigenetic marks and to study the effect of DNA sequence alterations (Zhou & Troyanskaya, 2015; Kelley *et al*, 2016).

## Early applications of neural networks in regulatory genomics

The first successful applications of neural networks in regulatory genomics replaced a classical machine learning approach with a deep model, without changing the input features. For example, Xiong *et al* (2015) considered a fully connected feedforward neural network to predict the splicing activity of individual exons. The model was trained using more than 1,000 pre‐defined features extracted from the candidate exon and adjacent introns. Despite the relatively low number of 10,700 training samples in combination with the model complexity, this method achieved substantially higher prediction accuracy of splicing activity compared to simpler approaches, and in particular was able to identify rare mutations implicated in splicing misregulation.

## Convolutional designs

More recent work using convolutional neural networks (CNNs) allowed direct training on the DNA sequence, without the need to define features (Alipanahi *et al*, 2015; Zhou & Troyanskaya, 2015; Angermueller *et al*, 2016; Kelley *et al*, 2016). The CNN architecture allows to greatly reduce the number of model parameters compared to a fully connected network by applying convolutional operations to only small regions of the input space and by sharing parameters between regions. The key advantage resulting from this approach is the ability to directly train the model on larger sequence windows (Box 2; Fig 2B).

**Box 2: Convolutional Neural Network**

Convolutional neural networks (CNNs) were originally inspired by cognitive neuroscience and Hubel and Wiesel's seminal work on the cat's visual cortex, which was found to have simple neurons that respond to small motifs in the visual field, and complex neurons that respond to larger ones (Hubel & Wiesel, 1963, 1970).

CNNs are designed to model input data in the form of multidimensional arrays, such as two‐dimensional images with three colour channels (LeCun *et al*, 1989; Jarrett *et al*, 2009; Krizhevsky *et al*, 2012; Zeiler & Fergus, 2014; He *et al*, 2015; Szegedy *et al*, 2015a) or one‐dimensional genomic sequences with one channel per nucleotide (Alipanahi *et al*, 2015; Wang *et al*, 2015; Zhou & Troyanskaya, 2015; Angermueller *et al*, 2016; Kelley *et al*, 2016). The high dimensionality of these data (up to millions of pixels for high‐resolution images) renders training a fully connected neural network challenging, as the number of parameters of such a model would typically exceed the number of training data to fit them. To circumvent this, CNNs make additional assumptions on the structure of the network, thereby reducing the effective number of parameters to learn.

A convolutional layer consists of multiple maps of neurons, so‐called feature maps or filters, with their size being equal to the dimension of the input image (panel A). Two concepts allow reducing the number of model parameters: local connectivity and parameter sharing. First, unlike in a fully connected network, each neuron within a feature map is only connected to a local patch of neurons in the previous layer, the so‐called receptive field. Second, all neurons within a given feature map share the same parameters. Hence, all neurons within a feature map scan for the same feature in the previous layer, however at different locations. Different feature maps might, for example, detect edges of different orientation in an image, or sequence motifs in a genomic sequence. The activity of a neuron is obtained by computing a discrete convolution of its receptive field, that is computing the weighted sum of input neurons, and applying an activation function (panel B).

In most applications, the exact position and frequency of features is irrelevant for the final prediction, such as recognizing objects in an image. Using this assumption, the pooling layer summarizes adjacent neurons by computing, for example, the maximum or average over their activity, resulting in a smoother representation of feature activities (panel C). By applying the same pooling operation to small image patches that are shifted by more than one pixel, the input image is effectively down‐sampled, thereby further reducing the number of model parameters.

A CNN typically consists of multiple convolutional and pooling layers, which allows learning more and more abstract features at increasing scales from small edges, to object parts, and finally entire objects. One or more fully connected layers can follow the last pooling layer (panel A). Model hyper‐parameters such as the number of convolutional layers, number of feature maps or the size of receptive fields are application‐dependent and should be strictly selected on a validation data set.

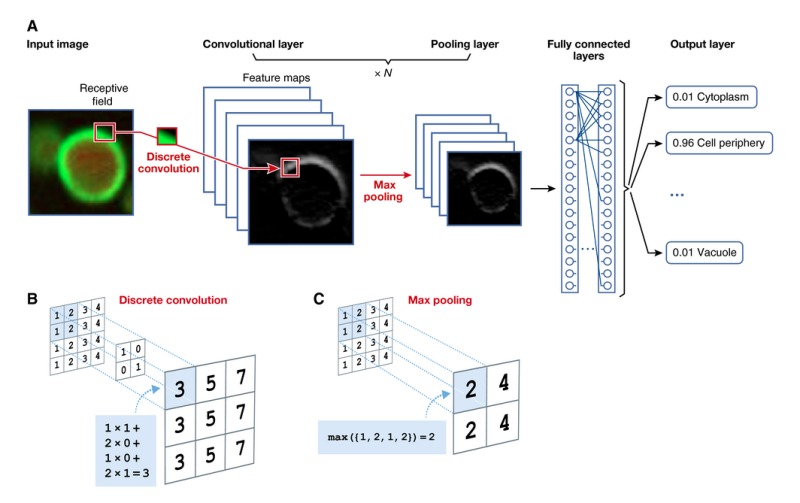

Alipanahi *et al* (2015) considered convolutional network architectures to predict specificities of DNA‐ and RNA‐binding proteins. Their *DeepBind* model outperformed existing methods, was able to recover known and novel sequence motifs, and could quantify the effect of sequence alterations and identify functional SNVs. A key innovation that enabled training the model directly on the raw DNA sequence was the application of a one‐dimensional convolutional layer. Intuitively, the neurons in the convolutional layer scan for motif sequences and combinations thereof, similar to conventional position weight matrices (Stormo *et al*, 1982). The learning signal from deeper layers informs the convolutional layer which motifs are the most relevant. The motifs recovered by the model can then be visualized as heatmaps or sequence logos (Fig 2D).

## *In silico* prediction of mutation effects

An important application of deep neural networks trained on the raw DNA sequence is to predict the effect of mutations *in silico*. Such model‐based assessments of the effect of sequence changes complement methods based on QTL mapping, and can in particular help to uncover regulatory effects of rare SNVs or to fine‐map likely causal genes. An intuitive approach for visualizing such predicted regulatory effects is mutation maps (Alipanahi *et al*, 2015), whereby the effect of all possible mutations for a given input sequence is represented in a matrix view (Fig 2E). The authors could further reliably identify deleterious SNVs by training an additional neural network with predicted binding scores for a wild‐type and mutant sequence (Fig 2C).

## Joint prediction of multiple traits and further extensions

Following their initial successes, convolutional architectures have been extended and applied to a range of tasks in regulatory genomics. For example, Zhou and Troyanskaya (2015) considered these architectures to predict chromatin marks from DNA sequence. The authors observed that the size of the input sequence window is a major determinant of model performance, where larger windows (now up to 1 kb) coupled with multiple convolutional layers enabled capturing sequence features at different genomic length scales. A second innovation was to use neural network architectures with multiple output variables (so‐called multitask neural networks) to predict multiple chromatin states in parallel. Multitask architectures allow learning shared features between outputs, thereby improving generalization performance, and markedly reducing the computational cost of model training compared to learning independent models for each trait (Dahl *et al*, 2014).

In a similar vein, Kelley *et al* (2016) developed the open‐source deep learning framework *Basset*, to predict DNase I hypersensitivity across multiple cell types and to quantify the effect of SNVs on chromatin accessibility. Again, the model improved prediction performance compared to conventional methods and was able to retrieve both known and novel sequence motifs that are associated with DNase I hypersensitivity. A related architecture has also been considered by Angermueller *et al* to predict DNA methylation states in single‐cell bisulphite sequencing studies (Angermueller *et al*, 2016). This approach combined convolutional architectures to detect informative DNA sequence motifs with additional features derived from neighbouring CpG sites, thereby accounting for methylation context. Most recently, Koh, Pierson and Kundaje applied CNNs to de‐noise genomewide chromatin immunoprecipitation followed by sequencing data in order to obtain a more accurate prevalence estimate for different chromatin marks (Koh *et al*, 2016).

At present, CNNs are among the most widely used architectures to extract features from fixed‐size DNA sequence windows. However, alternative architectures could also be considered. For example, recurrent neural networks (RNNs) are suited to model sequential data (Lipton, 2015) and have been applied for modelling natural language and speech (Hinton *et al*, 2012; Graves *et al*, 2013; Sutskever *et al*, 2014; Che *et al*, 2015; Deng & Togneri, 2015; Xiong *et al*, 2016), protein sequences (Agathocleous *et al*, 2010; Sønderby & Winther, 2014), clinical medical data (Che *et al*, 2015; Lipton *et al*, 2015) and to a limited extent DNA sequences (Xu *et al*, 2007; Lee *et al*, 2015). RNNs are appealing for applications in regulatory genomics, because they allow modelling sequences of variable length, and to capture long‐range interactions within the sequence and across multiple outputs. However, at present, RNNs are more difficult to train than CNNs, and additional work is needed to better understand the settings where one should be preferred over the other.

Complementary to supervised methods, unsupervised deep learning architectures learn low‐dimensional feature representations from high‐dimensional unlabelled data, similarly to classical principal component analysis or factor analysis, but using a nonlinear model. Examples of such approaches are stacked autoencoders (Vincent *et al*, 2010), restricted Boltzmann machines and deep belief networks (Hinton *et al*, 2006). The learnt features can be used to visualize data or as input for classical supervised learning tasks. For example, sparse autoencoders have been applied to classify cancer cases using gene expression profiles (Fakoor *et al*, 2013) or to predict protein backbones (Lyons *et al*, 2014). Restricted Boltzmann machines can also be used for unsupervised pre‐training of deep networks to subsequently train supervised models of protein secondary structures (Spencer *et al*, 2015), disordered protein regions (Eickholt & Cheng, 2013) or amino acid contacts (Eickholt & Cheng, 2012). Skip‐gram neural networks have been applied to learn low‐dimensional representations of protein sequences and improve protein classification (Asgari & Mofrad, 2015). In general, unsupervised models are a powerful approach if large quantities of unlabelled data are available to pre‐train complex models. Once trained, these models can help to improve performance on classification tasks, for which smaller numbers of labelled examples are typically available.

## Deep learning for biological image analysis

Historically, perhaps the most important successes of deep neural networks have been in image analysis. Deep architectures trained on millions of photographs can famously detect objects in pictures better than humans do (He *et al*, 2015). All current state‐of‐the‐art models in image classification, object detection, image retrieval and semantic segmentation make use of neural networks.

The convolutional neural network (Box 2) is the most common network architecture for image analysis. Briefly, a CNN performs pattern matching (convolution) and aggregation (pooling) operations (Box 2). At a pixel level, the convolution operation scans the image with a given pattern and calculates the strength of the match for every position. Pooling determines the presence of the pattern in a region, for example by calculating the maximum pattern match in smaller patches (max‐pooling), thereby aggregating region information into a single number. The successive application of convolution and pooling operations is at the core of most network architectures used in image analysis (Box 2).

## First applications in computational biology—pixel‐level classification

The early applications of deep networks for biological images focused on pixel‐level tasks, with additional models building on the network outputs. For example, Ning *et al* (2005) applied convolutional neural networks in a study that predicted abnormal development in *C. elegans* embryo images. They trained a CNN on 40 × 40 pixel patches to classify the centre pixel to cell wall, cytoplasm, nucleus membrane, nucleus or outside medium, using three convolutional and pooling layers, followed by a fully connected output layer. The model predictions were then fed into an energy‐based model for further analysis. CNNs have outperformed standard methods, for example Markov random fields and conditional random fields (Li, 2009) in such raw data analysis tasks, for example restoring noisy neural circuitry images (Jain *et al*, 2007).

Adding layers allows moving from clearing up pixel noise to modelling more abstract image features. Ciresan *et al* (2013) used five convolutional and pooling layers, followed by two fully connected layers, to find mitosis in breast histology images. This model won the mitosis detection challenge at the International Conference of Pattern Recognition 2012, outperforming competitors by a substantial margin. The same approach was also used to segment neuronal structures in electron microscopy images, classifying each pixel as membrane or non‐membrane (Ciresan *et al*, 2012). In these applications, while the CNNs were trained in an end‐to‐end manner, additional post‐processing was required to obtain class probabilities from the outputs for new images.

Successive pooling operations lose information on localization, as only summaries are retained from larger and larger regions. To avoid this, skip links can be added to carry information from early, fine‐grained layers forward to deeper ones. The currently best‐performing pixel‐level classification method for neuronal structures (*U‐Net;* Ronneberger *et al*, 2015) employs an architecture in which neurons take inputs from lower layers to localize high‐resolution features, as well as to overcome the arbitrary choice of context size.

## Analysis of whole cells, cell populations and tissues

In many cases, pixel‐level predictions are not required. For example, Xu *et al* directly classified colon histopathology images into cancerous and non‐cancerous, finding that supervised feature learning with deep networks was superior to using handcrafted features (Xu *et al*, 2014). Pärnamaa and Parts used CNNs to classify pre‐segmented image patches of individual yeast cells carrying a fluorescent protein to different subcellular localization patterns (Pärnamaa & Parts, 2016). Again, deep networks outperformed methods based on traditional features. Further, Kraus *et al* combined the segmentation and classification tasks into a single architecture that can be learned end‐to‐end and applied the model to full resolution yeast microscopy images (Kraus *et al*, 2015). This approach allowed classifying entire images without performing segmentation as a pre‐processing step. CNNs have even been applied to count bacterial colonies in agar plates (Ferrari *et al*, 2015). Since the early de‐noising applications on the pixel level, the field has been moving towards end‐to‐end image analysis pipelines that make use of large bioimage data sets, and the representational power of CNNs.

## Reusing trained models

Training convolutional neural networks requires large data sets. While biological data acquisition can be expensive, this does not mean that deep neural networks cannot be used when millions of images are not available. Regardless of image source, lower levels of the network tend to capture similar signal (edges, blobs) that are not specific to the training data and the application, but instead recur in perceptual tasks in general. Thus, convolutional neural networks can reuse pictures from a similar domain to help with learning, or even be pre‐trained on other data, thereby requiring fewer images to fine‐tune the model for the task of interest. Indeed, Donahue *et al* (2013) and Razavian *et al* (2014) showed that features learned from millions of images to classify objects, can successfully be used in image retrieval, detection or classification on new domains where only hundreds of images are labelled. The effectiveness of such an approach depends on the similarity between the training data and the new domain (Yosinski *et al*, 2014).

**Figure 3:**
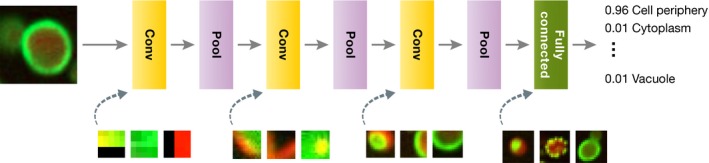
Convolution and pooling operators are stacked, thereby creating a deep network for image analysis In standard applications, convolution layers are followed by a pooling layer (Box 2). In this example, the lowest level convolutional units operate on 3 × 3 patches, but deeper ones use and capture information from larger regions. These convolutional pattern‐matching layers are followed by one or multiple fully connected layers to learn which features are most informative for classification. For each layer with learnable weights, three example images that maximize some neuron output are shown.

The concept of transferring model parameters has also been successful in bioimage analysis. For example, Zhang *et al* (2015) showed that features learned from natural images can be transferred to biological data, improving the prediction of *Drosophila melanogaster* developmental stages from *in situ* hybridization images. The model was first pre‐trained on data from the ImageNet (Russakovsky *et al*, 2015), an open corpus of more than one million diverse images, to extract rich features at different scales. Xie *et al* (2015) further used synthetic images to train a CNN for automatic cell counting in microscopy images. We expect that network repositories that host pre‐trained models will emerge for biological image analysis; such efforts already exist for general image processing tasks (see learning section below). These trained models could be downloaded and used as feature extractors (Fig 3), or further fine‐tuned and adapted to a particular task on small‐scale data.

## Interpreting and visualizing convolutional networks

Convolutional neural networks have been successful across many domains. In interpreting their performance, it is useful to understand the features they capture.

### Visualizing input weights

**Figure 4:**
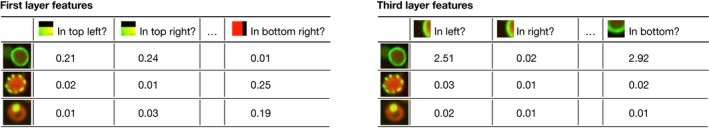
A pre‐trained network can be used as a generic feature extractor Feeding input into the first layer (left) gives a low‐level feature representation in terms of patterns (left to right) present in smaller patches in every cell (top to bottom). Neuron activations extracted from deeper layers (right) give rise to more abstract features that capture information from a larger segment of the image.

One way to understand what a particular neuron represents is to look for inputs that maximally activate it. Under some mathematical constraints, these patterns are proportional to the incoming weights (see also Box 1). Krizhevsky *et al* visualized weights in the first convolutional layer (Krizhevsky *et al*, 2012) and found that these maximally activating patterns correspond to colour blobs, edges at different orientations and Gabor‐like filters (Fig 4). Gabor filters are widely used pre‐defined features in image analysis; neural networks rediscover them in a data‐driven way as a useful component of the image model. Higher layer weights can be visualized as well, but as the inputs are not pixels, their weights are more difficult to interpret.

### Finding images that maximize neuron activity

To understand the deeper layers in terms of input pixels, Girshick *et al* (2014) retrieved and Simonyan *et al* (2013) generated images that maximize the output of individual neurons (Fig 4). While this approach yields no explicit representation, it can provide an overview of the type of features that differentiate images with large neuron activity from all others. Such visualizations tend to show that second‐layer features combine edges from the first layer, thereby detecting corners and angles; deeper layer neurons activate for specific object parts (e.g. noses, eyes); and the deepest layers detect whole objects (e.g. faces, cars). It is complicated to hand‐engineer features that look specifically for noses, eyes or faces, but neural networks can learn these features solely from input–output examples.

### Hiding important image parts

To understand which image parts are important for determining the value of each feature, Zeiler and Fergus (2014) occluded images with smaller grey boxes. The parts that are most influential will drastically change the feature value when occluded. In a similar vein, Simonyan *et al* (2013) and Springenberg *et al* (2014) visualized which individual pixels make the most difference in the feature, and Bach, Binder and colleagues developed pixel relevance for individual classification decisions in a more general framework (Bach *et al*, 2015). This information can also be used for object localization or segmentation, as the sensitive image pixels usually correctly correspond to the true object. Kraus *et al* (2015) used this idea to effectively localize cells in large microscopy images.

### Visualizing similar inputs in two dimensions

Visualizing the CNN representations can help gauge what inputs get mapped to similar feature vectors, and hence understand what the model has learned. Donahue *et al* (2013) projected CNN features into two dimensions to show that each subsequent layer transforms data to be more and more separable by a linear classifier. In general, different CNN visualization methods show that higher layer features are more specific to the learning task, while low‐level features tend to capture general aspects of images, such as edges and corners.

## Off‐the‐shelf tools and practical considerations

### Deep learning frameworks

Deep learning frameworks have been developed to easily build neural networks from existing modules on a high level. The most popular ones are Caffe (Jia *et al*, 2014), Theano (Bastien *et al*, 2012), Torch7 (Collobert *et al*, 2011) and TensorFlow (Abadi *et al*, 2016; Rampasek & Goldenberg, 2016) (Table 1), which differ in modularity, ease of use and the way models are defined and trained.

**Table 1:** Overview of existing deep learning frameworks, comparing four widely used software solutions

	Caffe	Theano	Torch7	TensorFlow
Core language	C++	Python, C++	LuaJIT	C++
Interfaces	Python, Matlab	Python	C	Python
Wrappers		Lasagne, Keras, sklearn‐theano		Keras, Pretty Tensor, Scikit Flow
Programming paradigm	Imperative	Declarative	Imperative	Declarative
Well suited for	CNNs, Reusing existing models, Computer vision	Custom models, RNNs	Custom models, CNNs, Reusing existing models	Custom models, Parallelization, RNNs

Caffe (Jia *et al*, 2014) is developed by the Berkeley Vision and Learning Center and is written in C++. The network architecture is specified in a configuration file and models can be trained and used via command line, without writing code at all. Additionally, Python and MATLAB interfaces are available. Caffe offers one of the most efficient implementations for CNNs and provides multiple pre‐trained models for image recognition, making it well suited for computer vision tasks. As a downside, custom models need to be implemented in C++, which can be difficult. Additionally, Caffe is not optimized for recurrent architectures.

Theano (Bastien *et al*, 2012; Team *et al*, 2016) is developed and maintained by the University of Montreal and written in Python and C++. Model definitions follow a declarative instead of an imperative programing paradigm, which means that the user specifies what needs to be done, not in which order. A neural network is declared as a computational graph, which is then compiled to native code and executed. This design allows Theano to optimize computational steps and to automatically derive gradients—one of its main strengths. Consequently, Theano is well suited for building custom models and offers particularly efficient implementations for RNNs. Software wrappers such as Keras (https://github.com/fchollet/keras) or Lasagne (https://github.com/Lasagne/Lasagne) provide additional abstraction and allow building networks from existing components, and reusing pre‐trained networks. The major drawback of Theano is frequently long compile times when building larger models.

Torch7 (Collobert *et al*, 2011) was initially developed at the University of New York and is based on the scripting language LuaJIT. Networks can be easily built by stacking existing modules and are not compiled, hence making it more suited for fast prototyping than Theano. Torch7 offers an efficient CNN implementation and access to a range of pre‐trained models. A possible downside is the need of the user to be familiar with the LuaJIT scripting language. Also, LuaJIT is less suited for building custom recurrent networks.

TensorFlow (Abadi *et al*, 2016) is the most recent deep learning framework developed by Google. The software is written in C++ and offers interfaces to Python. Similar to Theano, a neural network is declared as a computational graph, which is optimized during compilation. However, the shorter compile time makes it more suited for prototyping. A key strength of TensorFlow is native support for parallelization across different devices, including CPUs and GPUs, and using multiple compute nodes on a cluster. The accompanying tool TensorBoard allows to conveniently visualize networks in a web browser and to monitor training progress, for example learning curves or parameter updates. At present, TensorFlow provides the most efficient implementation for RNNs. The software is recent and under active development; hence, only few pre‐trained models are currently available.

## Data preparation

Training data are key for every machine learning application. Since more data with informative features usually result in better performance, effort should be spent on collecting, labelling, cleaning and normalizing data.

### Required data set sizes

Most of the successful applications of deep learning have been in supervised learning settings, where sufficient labelled training samples are available to fit complex models. As a rule of thumb, the number of training samples should be at least as high as the number of model parameters, although special architectures and model regularization can help to avoid overfitting if training data are scarce (Bengio, 2012).

Central problems in regulatory genomics, for example predicting molecular traits from genotype, are limited in the number of training instances; hundreds to at most tens of thousands of training examples are typical. The strategy of considering sequence windows centred on the trait of interest (e.g. splice site, transcription factor binding site or epigenetic marks; see Fig 2A) is now a widely used approach and helps increasing the number of input–output pairs from a single individual.

In image analysis, data can be abundant, but manually curated and labelled training examples are typically difficult to obtain. In such instances, the training set can be augmented by scaling, rotating or cropping the existing images, an approach that also enhances robustness (Krizhevsky *et al*, 2012). Another strategy is to reuse a network that was pre‐trained on a large data set for image recognition [e.g. AlexNet (Krizhevsky *et al*, 2012), VGG (Simonyan & Zisserman, 2014), GoogleNet (Szegedy *et al*, 2015b) or ResNet (He *et al*, 2015)], and to fine‐tune its parameters on the data set of interest (e.g. microscopy images for a particular segmentation task). Such an approach exploits the fact that different data sets share important characteristics and features, such as edges or curves, which can be transferred between them. Caffe, Lasagne, Torch and to a limited extend TensorFlow provide repositories with pre‐trained models.

### Partitioning data into training, validation and test sets

**Figure 5:**
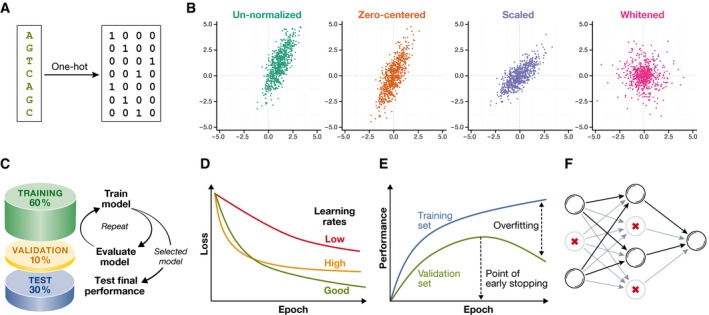
Data normalization for and pre‐processing for deep neural networks (A) DNA sequence one‐hot encoded as binary vectors using codes A = 1 0 0 0, G = 0 1 0 0, C = 0 0 1 0 and T = 0 0 0 1. (B) Continuous data (green) after zero‐centring (orange), scaling to unit variance (blue) and whiting (purple). (C) Holdout validation partitions the full data set randomly into training (~60%), validation (~10%) and test set (~30%). Models are trained with different hyper‐parameters on the training set, from which the model with the highest performance on the validation set is selected. The generalization performance of the model is assessed and compared with other machine learning methods on the test set. (D) The shape of the learning curve indicates if the learning rate is too low (red, shallow decay), too high (orange, steep decay followed by saturation) or appropriate for a particular learning task (green, gradual decay). (E) Large differences in the model performance on the training set (blue) and validation set (green) indicate overfitting. Stopping the training as soon as the validation set performance starts to drop (early stopping) can prevent overfitting. (F) Illustration of the dropout regularization. Shown is a feedforward neural network after randomly dropping out neurons (crossed out), which reduces the sensitivity of neurons to neurons in the previous layer due to non‐existent inputs (greyed edges).

Machine learning models need to be trained, selected and tested on independent data sets to avoid overfitting and assure that the model will generalize to unseen data. Holdout validation, partitioning the data into a training, validation and test sets, is the standard for deep neural networks (Fig 5C). The training set is used to learn models with different hyper‐parameters, which are then assessed on the validation set. The model with best performance, for example prediction accuracy or mean‐squared error, is selected and further evaluated on the test set to quantify the performance on unseen data and for comparison to other methods. Typical data set proportions are 60% for training, 10% for validation and 30% for model testing. If the data set is small, k‐fold cross‐validation or bootstrapping can be used instead (Hastie *et al*, 2005).

### Normalization of raw data

Appropriate choices for data normalization can help to accelerate training and the identification of a good local minimum.

Categorical features such as DNA nucleotides first need to be encoded numerically. They are typically represented as binary vectors with all but one entry set to zero, which indicates the category (*one‐hot coding*). For example, DNA nucleotides (categories) are commonly encoded as A = (1 0 0 0), G = (0 1 0 0), C = (0 0 1 0) and T = (0 0 0 1) (Fig 5A). A DNA sequence can then be represented as a binary string by concatenating the encoding nucleotides, and treating each nucleotide as an independent input feature of a feedforward neural network. In a CNN, the four bits of each encoded base are commonly considered analogously to colour channels of an image to preserve the entity of a nucleotide.

Numerical features are typically zero‐centred by subtracting their mean value. Image pixels are usually not zero‐centred individually, but jointly by subtracting the mean pixel intensity per colour channel. An additional common normalization step is to standardize features to unit variance. Whiting can be used to decorrelate features (Fig 5B), but can be computationally involved, since it requires computing the feature covariance matrix (Hastie *et al*, 2005). If the distribution of features is skewed due to a few extreme values, log transformations or similar processing steps may be appropriate. Validation and test data need to be normalized consistently with the training data. For example, features of the validation data need to be zero‐centred by subtracting the mean computed on the training data, not on the validation data.

## Model building

### Choice of model architecture

After preparing the data, design choices about the model architectures need to be made. The default architecture is a feedforward neural network with fully connected hidden layers, which is an appropriate starting point for many problems. Convolutional architectures are well suited for multi‐ and high‐dimensional data, such as two‐dimensional images or abundant genomic data. Recurrent neural networks can capture long‐range dependencies in sequential data of varying lengths, such as text, protein or DNA sequences. More sophisticated models can be built by combining different architectures. To describe the content of an image, for example, a CNN can be combined with an RNN, where the CNN encodes the image and the RNN generates the corresponding image description (Vinyals *et al*, 2015; Xu *et al*, 2015). Most deep learning frameworks provide modules for different architectures and their combinations.

### Determining the number of neurons in a network

The optimal number of hidden layers and hidden units is problem‐dependent and should be optimized on a validation set. One common heuristic is to maximize the number of layers and units without overfitting the data. More layers and units increase the number of representable functions and local optima, and empirical evidence shows that it makes finding a good local optimum less sensitive to weight initialization (Dauphin *et al*, 2014).

## Model training

The goal of model training is to find parameters *w* that minimize an objective function *L*(*w*), which measures the fit between the predictions the model parameterized by *w* and the actual observations. The most common objective functions are the cross‐entropy for classification and mean‐squared error for regression. Minimizing *L*(*w*) is challenging since it is high‐dimensional and non‐convex (Fig 5C); see also Box 1 and Fig 2.

### Stochastic gradient descent

Stochastic gradient descent is widely used to train deep models. Starting from an initial set of parameters *w*_*0*_, the gradient *dw* of *L* with respect to *w* is computed for a random batch of only few, for example 128, training samples. *dw* points to the direction of steepest descent, towards which *w* is updated with step size eta, the learning rate (Fig 1C). At each step, the parameters are updated into the direction of steepest descent until a minimum is reached, analogously to a ball running down a hill to a valley (Bengio, 2012). The training performance strongly depends on parameter initialization, learning rate and batch size.

### Parameter initialization

In general, model parameters should be initialized randomly to avoid local optima determined by a fixed initialization. Starting points for model parameters can be sampled independently from a normal distribution with small variance, or more commonly from a normal distribution with its variance scaled inversely by the number of hidden units in the input layer (Glorot & Bengio, 2010; He *et al*, 2015).

### Learning rate and batch size

The learning rate and batch size of stochastic gradient descent need to be chosen with care, since they can strongly impact training speed and model performance. Different learning rates are usually explored on a logarithmic scale such as 0.1, 0.01 or 0.001, with 0.01 as the recommended default value (Bengio, 2012). A batch size of 128 training samples is suitable for most applications. The batch size can be increased to speed up training or decreased to reduce memory usage, which can be important for training complex models on memory‐limited GPUs. The optimum learning rate and batch size are connected, with larger batch sizes typically requiring smaller learning rates.

### Learning rate decay

The learning rate can be gradually reduced during training, which is based on the idea that larger steps may be helpful in early training stages in order to overcome possible local optima, whereas smaller step sizes allow exploring narrow parameter regions of the loss function in advanced stages of training. Common approaches include to linearly reduce the learning rate by a constant factor such as 0.5 after the validation loss stops improving, or exponentially after every training iteration or epoch (Bengio, 2012; Gawehn *et al*, 2016).

### Momentum

Vanilla stochastic gradient descent can be extended by “momentum”, which usually improves training (Sutskever *et al*, 2013). Instead of updating the current parameter vector *w*_*t*_ at time *t* by the gradient vector *dw*_*t+1*_ directly, a fraction of the previous update is added to the current one. With momentum rate *v*, weights are updated by a momentum vector *m*_*t*+1_ = *ν* · *m*_*t*_ ‐ ∈ *dW*_*t*+1_. This approach can help to take larger steps in directions where gradients point consistently, and therefore speed up the convergence. The momentum rate *v* can be set between [0, 1], and a typical value is 0.9. Nesterov momentum (Nesterov, 1983, 2013) is a special form of the same concept, which sometimes provides additional advantages.

### Per‐parameter adaptive learning rate methods

To reduce the sensitivity to the specific choice of the learning rate, adaptive learning rate methods, such as RMSprop, Adagrad (Srivastava *et al*, 2014) and Adam (Kingma & Ba, 2014), have been developed in order to appropriately adapt the learning rate per parameter during training. The most recent method, Adam, combines the strengths of previous methods RMSprop and Adagrad and is generally recommended for many applications.

### Batch normalization

Batch normalization (Ioffe & Szegedy, 2015) is a recently described approach to reduce the dependency of training to the parameter initialization, speed up training and reduce overfitting. It is easy to implement, has marginal additional compute costs and has hence become common practice. Batch normalization zero centres and normalizes data not only at the input layer, but also at hidden layers before the activation function. This approach allows using higher learning rates and hence also accelerates training.

### Analysing the learning curve

To validate the learning process, the loss should be monitored as a function of the number of training epochs, that is the number of times the full training set has been traversed (Fig 5D). If the learning curve decreases slowly, the learning rate may be too small and should be increased. If the loss decreases steeply at the beginning but saturates quickly, the learning rate may be too high. Extreme learning rates can result in an increasing or fluctuating learning curve (Bengio, 2012).

### Monitoring training and validation performance

In parallel with the training loss, it is recommended to monitor the target performance such as the accuracy for both the training and validation set during training (Fig 5E). A low or decreasing validation performance relative to the training performance indicates overfitting (Bengio, 2012).

## Avoiding overfitting

Deep neural networks are notoriously difficult to train, and overfitting to data is a major challenge, since they are nonlinear and have many parameters. Overfitting results from a too complex model relative to the size of the training set, and can thus be reduced by decreasing the model complexity, for example the number of hidden layers and units, or by increasing the size of the training set, for example via data augmentation. The following training guidelines can help to avoid overfitting.

Dropout (Srivastava *et al*, 2014) is the most common regularization technique and often one of the key ingredients to train deep models. Here, the activation of some neurons is randomly set to zero (“dropped out”) during training in each forward pass, which intuitively results in an ensemble of different networks whose predictions are averaged (Fig 5E). The dropout rate corresponds to the probability that a neuron is dropped out, where 0.5 is a sensible default value. In addition to dropping out hidden units, input units can be dropped, however usually at a lower rate. Dropout is often combined with regularizing the magnitude or parameter values by the L2 norm, and less commonly the L1 norm.

Another popular regularization method is “early stopping”. Here, training is stopped as soon as the validation performance starts to saturate or deteriorate, and the parameters with the best performance on the validation set chosen.

Layerwise pre‐training (Bengio *et al*, 2007; Salakhutdinov & Hinton, 2012) should be considered if the model overfits despite the mentioned regularization techniques. Instead of training the entire network at once, layers are first pre‐trained unsupervised using autoencoders or restricted Boltzmann machines. Afterwards, the entire network is fine‐tuned using the actual supervised learning objective.

## Hyper‐parameter optimization

Table 2 summarizes recommendations and starting points for the most common hyper‐parameters, excluding architecture‐dependent hyper‐parameters such as the size and number of filters of a CNN. Since the best hyper‐parameter configuration is data‐ and application‐dependent, models with different configurations should be trained and their performance be evaluated on a validation set. As the number of configurations grows exponentially with the number of hyper‐parameters, trying all of them is impossible in practice (Bengio, 2012). It is therefore recommended to optimize the most important hyper‐parameters such as the learning rate, batch size or length of convolutional filters independently via line search, which is exploring different values while keeping all other hyper‐parameters constant. The refined hyper‐parameter space can then be further explored by random sampling, and settings with the best performance on the validation set are chosen. Frameworks such as Spearmint (Snoek *et al*, 2012), Hyperopt (Bergstra & Cox, 2013) or SMAC (Hutter *et al*, 2011) allow to automatically explore the hyper‐parameter space using Bayesian optimization. However, although conceptually more powerful, they are at present more difficult to apply and parallelize than random sampling.

**Table 2:** Central parameters of a neural network and recommended settings

Name	Range	Default value
Learning rate	0.1, 0.01, 0.001, 0.0001	0.01
Batch size	64, 128, 256	128
Momentum rate	0.8, 0.9, 0.95	0.9
Weight initialization	Normal, Uniform, Glorot uniform	Glorot uniform
Per‐parameter adaptive learning rate methods	RMSprop, Adagrad, Adadelta, Adam	Adam
Batch normalization	Yes, no	Yes
Learning rate decay	None, linear, exponential	Linear (rate 0.5)
Activation function	Sigmoid, Tanh, ReLU, Softmax	ReLU
Dropout rate	0.1, 0.25, 0.5, 0.75	0.5
L1, L2 regularization	0, 0.01, 0.001	

## Training on GPUs

Training neural networks is more time‐consuming compared to shallow models and can take hours, days or even weeks, depending on the size of training set and model architecture. Training on GPUs can considerably reduce the training time (commonly by tenfold or more) and is therefore crucial for evaluating multiple models efficiently. The reason for this speedup is that learning deep networks requires large numbers of matrix multiplications, which can be parallelized efficiently on GPUs. All state‐of‐the‐art deep learning frameworks provide support to train models on either CPUs or GPUs without requiring any knowledge about GPU programming. On desktop machines, the local GPU card can often be used if the framework supports the specific brand. Alternatively, commercial providers provide GPU cloud compute clusters.

## Pitfalls

No single method is universally applicable, and the choice of whether and how to use deep learning approaches will be problem‐specific. Conventional analysis approaches will remain valid and have advantages when data are scarce or if the aim is to assess statistical significance, which is currently difficult using deep learning methods. Another limitation of deep learning is the increased training complexity, which applies both to model design and the required compute environment.

## Conclusion

Deep learning methods are a powerful complement to classical machine learning tools and other analysis strategies. Already, these approaches have found use in a number of applications in computational biology, including regulatory genomics and image analysis. The first publicly available software frameworks have helped to reduce the overhead of model development and provided a rich, accessible toolbox to practitioners. We expect that continued improvement of software infrastructure will make deep learning applicable to a growing range of biological problems.

## Conflict of interest

The authors declare that they have no conflict of interest.
